# Scaling Laws for Mitotic Chromosomes

**DOI:** 10.3389/fcell.2021.684278

**Published:** 2021-06-11

**Authors:** Eric M. Kramer, P. A. Tayjasanant, Bethan Cordone

**Affiliations:** Department of Physics, Bard College at Simon’s Rock, Great Barrington, MA, United States

**Keywords:** chromosome, scaling analysis, spindle, vertebrate, angiosperm, mitosis

## Abstract

During mitosis in higher eukaryotes, each chromosome condenses into a pair of rod-shaped chromatids. This process is co-regulated by the activity of several gene families, and the underlying biophysics remains poorly understood. To better understand the factors regulating chromosome condensation, we compiled a database of mitotic chromosome size and DNA content from the tables and figures of >200 published papers. A comparison across vertebrate species shows that chromosome width, length and volume scale with DNA content to the powers ∼1/4, ∼1/2, and ∼1, respectively. Angiosperms (flowering plants) show a similar length scaling, so this result is not specific to vertebrates. Chromosome shape and size thus satisfy two conditions: (1) DNA content per unit volume is approximately constant and (2) the cross-sectional area increases proportionately with chromosome length. Since viscous drag forces during chromosome movement are expected to scale with length, we hypothesize that the cross-section increase is necessary to limit the occurrence of large chromosome elongations that could slow or stall mitosis. Lastly, we note that individual vertebrate karyotypes typically exhibit a wider range of chromosome lengths as compared with angiosperms.

## Introduction

As somatic cells enter mitosis the genome is radically reorganized. Each chromosome is condensed into a pair of rod-shaped chromatids, conjoined throughout their length, and transported to the metaphase plate. At anaphase, the sister chromatids are physically separated and dragged towards opposite spindle poles by interactions between the kinetochores and the spindle microtubules. This process ensures that each daughter cell receives a full copy of the genome.

The last decade has seen rapid progress uncovering the gene families that regulate chromatin re-organization during mitosis. Perhaps the most important are condensins (Nasmyth, 2001; Alipour and Marko, 2012). These are ATPase complexes that bind chromatin, then act on the bound strand to initiate and grow a lateral loop. Several other gene families have also been shown to contribute to the maintenance of mitotic chromosome shape: cohesins link adjacent chromatin strands, topo-isomerases allow chromatin strands to pass through one another, histone kinases regulate histone-histone interactions, and Ki-67 coats the outside of chromosomes and maintains their spatial separation (Gerlich et al., 2006; Samejima et al., 2012; Wilkins et al., 2014; Kruitwagen et al., 2015; Cuylen et al., 2016; Nielsen et al., 2020). These proteins interact with chromatin in complex ways to maintain the chromosome in a highly dynamic state. A recent Hi-C study of human chromosomes revealed that the spatial organization of the genome inside a chromosome is undergoing constant change as chromatin loops form, grow, and interpenetrate (Gibcus et al., 2018). The detailed way in which the chromatin is folded and looped within the chromatids is a topic of current research, and many open questions remain (Daban, 2015; Piskadlo and Oliveira, 2016; Gibcus et al., 2018; Brahmachari and Marko, 2019).

It has been suggested by several authors that the gross features of the chromosomes—length, width, and volume—might be used to inform and constrain chromatin-scale models (Rothfels et al., 1966; Mora-Bermudez et al., 2007; Daban, 2014; Brahmachari and Marko, 2019). There are two sets of observations that any detailed theory of chromatin organization should account for. First, all the chromosomes in a dividing cell appear to have approximately the same width. This result appears in the classic cytogenetics text by Darlington (1937), and it has been confirmed by modern methods using human cell cultures (Sumner, 1991; Booth et al., 2016). Second, the lengths of the chromosomes in a dividing cell are approximately proportional to their DNA content (Mayall et al., 1984; Praca-Fontes et al., 2014; Lacerda et al., 2019). Both these observations have only been quantified in a small number of species, and should be regarded as preliminary, although they are known to hold true in human cells. In combination, these two rules suggest that chromosome volume is also proportional to DNA content. In other words, DNA density is approximately constant for the chromosomes in a dividing cell (Booth et al., 2016).

In addition to these static features, any chromatin-scale model of chromosomes should also account for their dynamics, especially the gradual contraction of chromosomes during mitosis. The shortening begins during late prophase, continues through metaphase, and reaches a minimum length as the chromatids separate in anaphase (Bajer, 1959; Yunis et al., 1978; Van Dyke et al., 1986). The contraction is believed to be tightly regulated, since the *relative* lengths of the chromosomes remain approximately constant as their absolute lengths decrease—a fact necessary to preserve the proportionality described in the previous paragraph. The decrease in chromosome length is compensated by an increase in width, so that the volume of each mitotic chromosome remains approximately constant (Darlington, 1937; Yunis et al., 1978; Sumner, 1991; Booth et al., 2016). A recent study of rat kidney culture cells shows that the chromosome volume holds steady during metaphase, and only fluctuates by ∼25% during the transition to anaphase (Mora-Bermudez et al., 2007).

In this paper we are interested in the ways the gross features of chromosomes vary between species. Considering the previous paragraphs, the reader might expect that the summed chromosome length in a dividing cell is approximately proportional to genome size. However, studies find that total chromosome length scales sub-linearly with genome size (Rothfels et al., 1966; Peruzzi et al., 2009; Hara et al., 2016). In other words, species with a larger genome tend to have mitotic chromosomes that are more heavily contracted (more DNA per unit length), with a correspondingly larger width (Daban, 2014). This inspired two recent proposals that chromosome length and width might obey universal scaling laws common to all metazoans, and perhaps even to all eukaryotes (Daban, 2014; Brahmachari and Marko, 2019). To date, evidence supporting these scaling laws remains sparse, and published analyses ignore the range of chromosome lengths present in a metaphase. Human autosomes, for example, range in length over a factor of 6× (Van Dyke et al., 1986), and in some species of reptiles and birds the range can be much larger (see, e.g., Hammar, 1970).

In this paper we present a database of metaphase chromosome sizes and DNA content in hundreds of species of vertebrates and angiosperms (flowering plants). These two clades have the following useful features in common. First, the genome sizes of species in both clades have been a topic of research for decades, and genome size databases already exist (Gregory, 2019; Leitch et al., 2019). Second, in both clades the total genome size ranges over several orders of magnitude, a fact which permits a meaningful scaling analysis to be performed. Third, the number and relative length of mitotic chromosomes—called the karyotype—is a standard datum in species identification and classification, so that detailed information is abundant in the literature. Thus, a comparative analysis within and between clades is possible.

We analyze the data to find scaling law relations between chromosome width, length, volume, and DNA content. We find nearly identical length scaling exponents for both vertebrates and angiosperms. After presenting our scaling analysis, we discuss our results in the larger context of chromosome biology and evolution.

## Materials and Methods

### Terminology and Notation

Mitotic chromosome lengths are often reported as a sum, typically called the total haploid or total diploid complement length. We use L_*tot*_ to denote the total diploid complement length of chromosomes, including both autosomes and heterosomes (measured in μm). In biology, diploid chromosome number is universally denoted by 2n, and diploid genome size by 2C (we use units of pg for C-values, although Mbp is also common (Dolezel et al., 2003)). Using this notation, the average chromosome length is L_*avg*_ = L_*tot*_/2n and the average DNA content per chromosome is c_*avg*_ = 4C/2n (each mitotic chromosome includes a pair of chromatids, so 2n chromosomes contain 4C DNA). For the majority of species in our database, we were also able to find the length of the longest chromosome in a metaphase complement, L_*max*_. The DNA content of the longest chromosome, c_*max*_, may be estimated using the technique we present in the section “Results.”

### Data Sources

We drew most values of vertebrate genome size from the Animal Genome Database compiled by Gregory (2019) (www.genomesize.com, version downloaded 2019) and angiosperm genome size from the Plant DNA C-values Database maintained by the Royal Botanic Gardens at Kew (cvalues.science.kew.org, version downloaded 2019; Leitch et al., 2019). Using these two databases as a starting point, we sought to match individual species to their corresponding chromosome data using internet searches with keywords like “karyotype,” “chromosomes,” and “cytology.” We were careful to select species covering a wide range of 2C and 2n values, and also to gather data for species covering a wide phylogenetic diversity.

In publications on plant cytology, it is common for authors to compile tables of chromosome lengths or total complement lengths. Most of our angiosperm entries come from such tables. In publications on vertebrates, however, length tabulations are much less common, so we typically drew data from published figures showing stained mitotic chromosomes, or from bar graphs showing the distribution of chromosome lengths. To measure chromosome dimensions from published figures, images were extracted from the PDF and imported to ImageJ (version 1.52a, download from imagej.nih.gov/ij/). Individual chromosomes were traced by hand using the segmented line tool.

For the majority of vertebrate species, chromosome widths were also determined. In ImageJ, we measured the apparent width of several large chromosomes in a figure and took the median value. We generally focused on portions of the chromosome away from the centromere, which often stains poorly, and away from the chromosome ends, which were often visibly thicker than the rest of the chromatid. We were also careful to avoid portions of chromosomes that bent out of the focal plane. In cases where chromatids were prematurely separated (this often happens in chemically arrested mitosis), we determined the width of one chromatid and doubled it for the database entry. It is a classical result that all chromosomes in a set are generally contracted by similar amounts, such that the widths are comparable across the karyotype (Darlington, 1937; Sumner, 1991; Booth et al., 2016). Our observations were consistent with this view, with the obvious exception of microchromosomes, which were excluded from our width determinations. We did not collect data on chromosome width in angiosperms because most of our plant data comes from tables rather than images.

In cases where a source reported more than one value for a quantity of interest, we recorded the median value in our database.

### Regressions

The regression techniques commonly used in scaling analysis assume that errors around the regression line are distributed normally. This assumption is problematic in an analysis of the sort conducted here, since individual outliers can skew the regression line. (For example, consider the possibility that a few papers in the database contain an error, e.g., the figure scale bars are twice too large.) To overcome this potential difficulty, we use Siegel’s technique of repeated medians (Siegel, 1982). This technique is insensitive to outliers and still gives results comparable to traditional regression in cases where the errors are normally distributed. To find a power-law fit, we take the log_10_ of both variables and provide these as input to the statistics package mblm (Komsta, 2019) in R (R Core Team, 2020). Package output includes slope, intercept, and 95% confidence intervals for the regression line. The slope of the regression line is our scaling exponent, and the intercept is log_10_(a) where a is the multiplicative constant.

## Results

### Vertebrates

To examine the relationship between mitotic chromosome size and DNA content, we compiled a database of chromosome length and genome size for 258 vertebrate species, spanning 109 families (Supplementary Table 1). Additional karyotype data—chromosome width and the length of the longest chromosome in a set—were also collected for the majority of species. We were careful to select species that adequately covered the known range of vertebrate C-values and chromosome numbers. The C-values in the database range from 0.34 to 115 pg, and the chromosome numbers range from 2n = 6 to 2n = 168. Additional details of the database may be found in Table 1.

**TABLE 1:** Database Information.

	**Vertebrate database**	**Angiosperm database**
number of entries	281	504
number of species	258	419
number of genera	181	120
number of families	109	39
smallest genome (1C, in pg)	0.34	0.16
largest genome (1C, in pg)	115	152
smallest chromosome number (2n)	6	4
largest chromosome number (2n)	168	104
number of references	112	115

**FIGURE 1:**
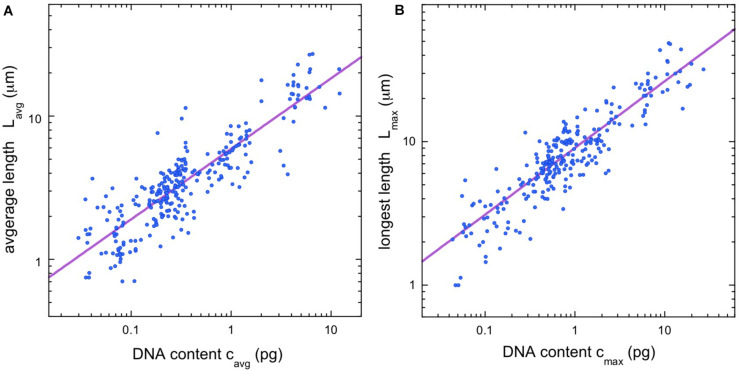
Chromosome length scaling in vertebrates. **(A)** Average chromosome length vs average chromosomal DNA content. The regression line (violet) corresponds to a power law, L_*avg*_ = a_*Lavg*_ (c_*avg*_)^α^, with the constant a_*Lavg*_ = 5.93 and the exponent α = 0.492 (95% confidence intervals 5.81–6.16 and 0.480–0.510, respectively, *N* = 281). **(B)** Length of longest chromosome vs. estimated DNA content. The regression line (violet) corresponds to a power law, L_*max*_ = a_*Lmax*_ (c_*max*_)^α^, with the constant a_*Lmax*_ = 9.07 and the exponent α = 0.465 (95% confidence intervals 8.80–9.22 and 0.458–0.484 respectively, *N* = 256). All regressions calculated using Siegel’s repeated medians (Siegel, 1982), a nonparametric technique that is insensitive to outliers.

Using the database, it is straightforward to calculate the average chromosome length (L_*avg*_ = L_*tot*_/2n) and the average DNA content per chromosome (c_*avg*_ = 4C/2n). Figure 1A shows a double-logarithmic plot of the resulting data. The data cluster around a straight line, which suggests a power-law dependence, L_*avg*_ = a_*Lavg*_ (c_*avg*_)^α^. Using nonparametric statistics, we find a scaling exponent of α = 0.492, with confidence intervals listed in the figure legend.

Since our values for average length and average DNA content both depend on chromosome number, we had some concerns that species with a proliferation of small chromosomes might skew our results. In particular, many bird and reptile species have a large numbers of “microchromosomes”—chromosomes so small that their length cannot be adequately resolved in an optical microscope (see, e.g., Hammar, 1970). Thus, we sought a second way to quantify chromosome scaling that would be independent of the total number of chromosomes. For most of the entries in the vertebrate database (256/281), we were able to gather data on the length of the longest chromosome for each metaphase, L_*max*_. We also needed an estimate for the DNA content of the longest chromosome. While the DNA content for most species is only available as a total, we can estimate the DNA content in any one chromosome using the cytogenetic rule discussed in the introduction: namely, that the relative lengths of the chromosomes in a dividing cell are proportional to their relative DNA content. This rule seems to be especially robust for the longest chromosomes in a karyotype. For example, in humans, the longest chromosome contributes 9% of the total haploid autosomal length (Van Dyke et al., 1986), while modern genomic methods indicate that it contains 8.6% of the total autosomal DNA (ensemble release 87, Aken et al., 2017). Thus, for each database entry where a longest chromosome was available, we estimated the DNA content of that chromosome as

(1) $${\text{c}}_{\text{max}}=4\,C\,\left({\text{L}}_{\text{max}}/{\text{L}}_{\text{tot}}\right).$$

Figure 1B shows a plot of L_*max*_ vs. c_*max*_. The scaling exponent is just 5% lower than that found for the average quantities, α = 0.465, with confidence intervals listed in the figure legend. The confidence intervals of the two scaling exponents have some overlap around α ≈ 0.48, although there is no *a priori* reason to assume the two exponents should be identical.

**FIGURE 2:**
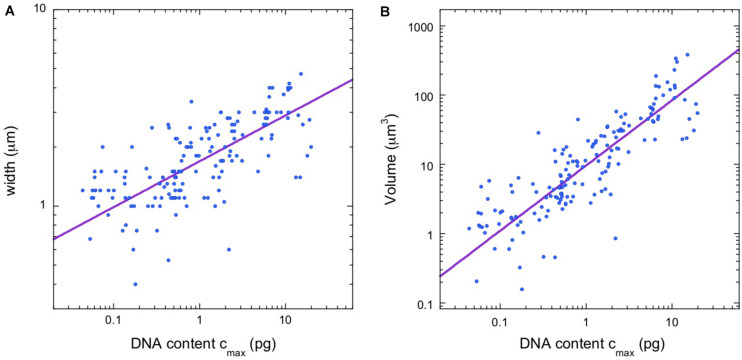
Additional vertebrate scaling results. **(A)** Chromosome width vs. estimated DNA content for longest chromosome. The regression line (violet) is a power law, w = a_*w*_ (c_*max*_)^β^, with the constant a_*w*_ = 1.68 and the exponent β = 0.234 (95% confidence intervals 1.64–1.74 and 0.211–0.243, respectively, *N* = 158). **(B)** Estimated volume of longest chromosome vs. estimated DNA content. The regression line (violet) is a power law, V = a_*V*_ (c_*max*_)^γ^, with the constant a_*V*_ = 9.55 and the exponent γ = 0.942 (95% confidence intervals 9.09–10.33 and 0.877–0.960, respectively, *N* = 158).

After identifying a scaling law for chromosome lengths, we considered the possibility of a scaling law for chromosome width as well. We were able to gather data on chromosome width for the majority (158/281) of vertebrate species in the database. Figure 2A shows a plot of width vs. c_*max*_, where we use the DNA content of the largest chromosomes, rather than the average values, to avoid complications due to microchromosomes. A power-law fit, w = a_*w*_ (c_*max*_)^β^, finds a scaling exponent β = 0.234, with confidence intervals in the figure legend. The availability of chromosome width data also allowed us to estimate the volume of the longest chromosome in the karyotype, V = 2πL_*max*_(w/4)^2^, which is the volume of a pair of adjacent, cylindrical chromatids of length L_*max*_ and cylinder radius w/4. Figure 2B shows a power-law fit, V = a_*V*_ (c_*max*_)^γ^. The scaling exponent is γ = 0.942, with confidence intervals in the figure legend. Since our equation for the chromosome volume relates it to L_*max*_ and w, we expect γ ≈ α + 2β = 0.933, and this is indeed the case.

### Flowering Plants

We compiled a database of angiosperm mitotic chromosome length and genome size (Supplementary Table 2). The data includes 419 species and spans 39 families of basal angiosperms, monocots, and eudicots. Genome size ranges over three orders of magnitude, from 0.16 to 152 pg, and chromosome number ranges from 2n = 4 to 2n = 104. Additional information about the angiosperm database may be found in Table 1.

**FIGURE 3:**
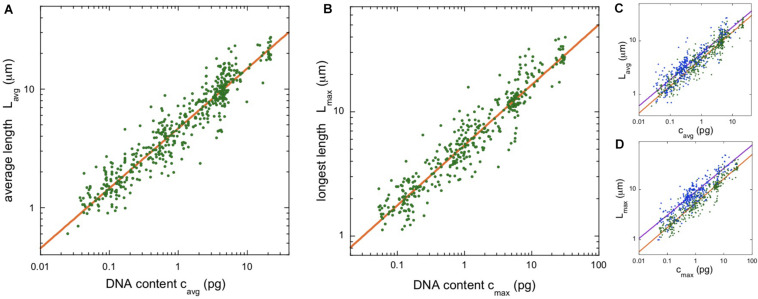
Chromosome length scaling in angiosperms (flowering plants). **(A)** Average chromosome length vs average chromosomal DNA content. The regression line (orange) corresponds to a power law, L_*avg*_ = a_*Lavg*_ (c_*avg*_)^α^, with the constant a_*Lavg*_ = 4.64 and the exponent α = 0.506 (95% confidence intervals 4.61–4.72 and 0.498–0.509, respectively, *N* = 504). **(B)** Length of longest chromosome vs estimated DNA content. The regression line (orange) corresponds to a power law, L_*max*_ = a_*Lmax*_ (c_*max*_)^α^, with the constant a_*Lmax*_ = 5.37 and the exponent α = 0.485 (95% confidence intervals 5.33–5.46 and 0.479–0.491, respectively, *N* = 380). **(C,D)** Overlay of the length scaling plots for vertebrates (blue) and angiosperms (green).

Figure 3A shows a plot of average chromosome length vs average chromosomal DNA content for the angiosperm data. The scaling exponent is α = 0.506, with confidence intervals listed in the figure legend.

To parallel our analysis in vertebrates, we found the length of the longest mitotic chromosome in the karyotype, L_*max*_, for the majority (380/504) of angiosperm entries. To estimate the DNA content of the longest chromosome, we again used Eq. (1). The rule that relative chromosome lengths at metaphase are linearly proportional to relative DNA content also seems to apply in plants. In *Pisum sativum* (pea), the longest chromosome is 17.4% of the total haploid complement length, and it contains 17.5% of the genome (Praca-Fontes et al., 2014). Our data are plotted in Figure 3B. The scaling exponent, α = 0.485, is just 4% lower than that found for the average quantities, but the 95% confidence intervals for the exponents do not overlap.

## Discussion

### The Scaling Laws

We used power-laws to quantify the dependence of mitotic chromosome length, width, and volume on chromosome DNA content. For vertebrates, we found scaling exponents α = 0.49 and 0.46, and for angiosperms, 0.51 and 0.48, depending on the details of the analysis. The median of these is 0.485, and we approximate this as α ≈ 0.5 in the following discussion.

The constants of proportionality in the scaling laws are also interesting to compare. All four constants fall within a factor of 2 of each other. In other words, the scaling observed for vertebrates and angiosperms is not just characterized by a similar exponent, but by qualitative similarities in the absolute chromosome lengths as well (see Figures 3C,D). Although qualitatively similar, the differences between the clades is statistically significant, with vertebrates having longer chromosomes than angiosperms. This is most apparent for the largest chromosomes in a karyotype, as illustrated in Figure 3D. The constant of proportionality, a_*Lmax*_, is 67% larger in vertebrates than in angiosperms and the confidence intervals do not overlap. The fact that animal chromosomes tend to be less compact than plant chromosomes has been noted previously (Greilhuber, 1977).

Another interesting difference between vertebrates and angiosperms is the degree to which chromosome lengths in a karyotype vary between smallest and largest. Although we did not collect data on the smallest chromosomes in a metaphase (limits on the resolution of optical microscopes made this problematic), we were able to use the ratio L_*max*_/L_*avg*_ to characterize the spread of lengths in a karyotype. Less than 3% (10/380) of the entries in our angiosperm database have L_*max*_/L_*avg*_ > 2, while fully 50% (127/254) of our vertebrate entries satisfy this condition. In other words, vertebrate karyotypes show a wider spread of chromosome lengths than angiosperm karyotypes. Two illustrative examples: in humans, the length ratio of longest to shortest autosomes is 6.4 (Van Dyke et al., 1986) while in *P. sativum* (pea) the ratio is 1.5 (Praca-Fontes et al., 2014).

In vertebrates, we were also able to examine the dependence of chromosome width and volume on DNA content. The width of vertebrate chromosomes scales with DNA content to the exponent β = 0.234, a value we approximate as β ≈ 0.25 in the following discussion. The volume is nearly proportional to the DNA content (scaling exponent γ = 0.94), as expected if DNA density is approximately uniform across vertebrate species (γ ≈ 1). Examining the range of DNA density values in our database (Supplementary Table 2), we find a median of 0.093 pg/μm^3^, with 25th and 75th percentiles 0.058 and 0.171 pg/μm^3^ respectively. This range overlaps with previous estimates for human chromosomes (Daban, 2000).

These observations raise the questions of why the scaling exponents have these values, and why chromosome lengths should be so similar between two clades that diverged 1.6 billion years ago (Wang et al., 1999). Flowering plants and vertebrates have in common most of the major molecular players in chromatin remodeling and condensation, including topoisomerases, cohesins, and condensins (Hirano, 2005; Forterre et al., 2007; Yuan et al., 2011), so it seems probable that the similarity in the scaling reflects a shared feature of the biochemistry and biophysics of chromatin condensation, or perhaps of mitosis more generally.

### Previous Scaling Results

The idea that a cross-species comparison of mitotic chromosome size might clarify the dynamics of chromosome condensation dates back to at least 1966 (Rothfels et al., 1966), and has seen a renaissance in the last decade. A brief survey will show the range of approaches:

Daban (2014) presented a theoretical model of a chromatid as a cylindrical stack of thin chromatin plates. To help quantify the parameters of his model, he surveyed chromosome size in 8 species of plants and animals. Based on this small sample, he made the approximations that chromosome aspect ratio (L/w) and chromosome DNA density are both approximately constant. This led to predictions for the scaling exponents, α = β = 1/3.

Hara et al. (2016) surveyed chromosome and nuclear size in ∼10 species of vertebrates, invertebrates, and plants. They reported scaling laws for the total complement length L_*tot*_, but did not consider the chromosomes individually. They explained their scaling results by arguing that the chromosomes must pack efficiently into the area of a thin metaphase plate.

Brahmachari and Marko (2019) made a theoretical treatment of chromosome condensation from the perspective of polymer physics. They focused on biophysical constraints like the breaking strength of DNA and the need for chromosomes at the metaphase plate to disentangle before anaphase. Their predicted scaling exponent for the length was α = 0.44, and their exponent for the width fell in the range β = 0.28−0.50, depending on the assumptions made about the activity of cohesins and condensins.

### Scaling for an Elastic Chromatid in a Viscous Medium

We hypothesize that the length and width scaling may be explained by considering the elastic response of a chromatid to the forces that act on it. During mitosis the chromosomes are dragged into the metaphase plate by interactions with the microtubules (MTs) of the spindle, and during anaphase the chromatids are separated and dragged towards opposite poles of the dividing cell. Early measurements based on the Brownian motion of particles much smaller than chromatids implied that the viscosity of the cytoplasm was in the range 0.2–10 Poise, not enough to bend or stretch chromatids during these movements (Niklas, 1965; Taylor, 1965). However, observations of in-line strain exhibited by chromatids are as high as 16% (Alexander and Rieder, 1991), and chromatids routinely stretch into arcs or J-shapes as they are dragged towards the spindle poles (see, e.g., Bajer, 1959). The resolution of this paradox appears to be the action of a much larger drag force exerted by the MT network of the spindle itself, experienced by any object that is too large to pass through the gaps in the network (Houchmandzadeh et al., 1997; Shimamoto et al., 2011). The spindle creates an effective viscosity of ∼1,000 Poise in the chromosome microenvironment, which is easily capable of stretching and bending chromatids.

We propose that the elastic properties of a chromosome must be adequate to sustain these large drag forces without undergoing large strains (>100%). The disadvantage of large strains isn’t chromosome breakage—experiments on chromosomes have shown that they can undergo strains of 100% or more and still return to their unstretched length (Poirier et al., 2000). Rather, since the distance between the spindle poles and the midplane is often comparable to the length of the chromosomes (see, e.g., Bajer and Mole-Bajer, 1956), large strains at anaphase can be associated with a range of mitotic abnormalities where one or more chromatids are slow to exit the metaphase plate, or in extreme cases do not exit at all. If chromosomes do not clear the division midplane during anaphase, it can trigger an abscission checkpoint (sometimes called the NoCut checkpoint) that stalls division (Norden et al., 2006; Amaral et al., 2016). Chromosomes that fail to fully segregate to a spindle pole can also delay the reconstitution of the nuclear envelope (Afonso et al., 2014). In other words, large chromosome strains may cause the delay or failure of cytokinesis.

In a high-viscosity environment, the drag force on elongated bodies is approximately proportional to their length (Niklas, 1965; Ui et al., 1984). To support this force without sustaining large strains, we propose that longer chromosomes must have a proportionately larger cross-section. If we approximate a chromatid as an elastic rod, the force required to stretch a chromosome by 100% (sometimes called the stretching modulus) may be written *F*_100_ = *YA*, where *Y* is the effective Young’s modulus of the condensed chromatin and *A* is the cross-sectional area (Niklas, 1983). If the degree of molecular connection between chromatin strands is comparable among chromosomes of different size, then *Y* will be approximately constant and *F*_100_ will scale with cross sectional area. Experiments on isolated chromatids indeed find that *Y* is approximately independent of chromosome size (Niklas, 1983; Houchmandzadeh et al., 1997; Houchmandzadeh and Dimitrov, 1999). Thus, the assumption that *F*_100_ is proportional to the cross section is consistent with measurements.

This force balance argument therefore implies a simple scaling between chromosome length and cross-sectional area, L ∼ w^2^. In terms of the scaling exponents,

(2) α=2⁢β

If we also assume that the DNA density of chromosomes is approximately constant, we have (DNA content) ∼ (Chromosome Volume) ∼ L w^2^. In terms of the scaling exponents,

(3) α+2⁢β= 1

The solution to Eqs 2 and 3 is α = 1/2 and β = 1/4.

We suggest that evolution has shaped chromosomes in this way by varying the dose and the activity of gene products with a role in condensation. Individuals with too little condensation (longer mitotic chromosomes, with a smaller width) would tend to suffer from delayed or incomplete mitosis, as described above. Conversely, individuals with too much condensation (i.e., the length contracts more than the scaling law prescription) would presumably suffer a competitive disadvantage since condensation is time-consuming and energetically costly (Salazar-Roa and Malumbres, 2017).

Our scaling hypothesis is preliminary, but it is consistent with the lack of chromosome condensation observed in some lower eukaryotes. One particularly well-studied example is the budding yeast *Saccharomyces cerevisiae*. Optical and electron microscopy do not reveal any rodlike chromatin during mitosis (Peterson and Ris, 1976), and less direct probes suggest that mitotic chromosomes are only contracted by a factor of ∼2 compared with the interphase arrangement (Guacci et al., 1994; Vas et al., 2007). We propose that the lack of condensation is related to the sparse distribution of MTs in the yeast spindle (Winey et al., 1995): just one MT connects each chromatid to a spindle pole, and most of the remainder lie in a rodlike bundle of ∼8 MTs that stretches between the poles. It therefore seems likely that spindle-mediated drag on the chromatids is weak or absent. The co-occurrence in yeast of limited chromosome condensation and sparse spindle MTs are consistent with our argument.

## Conclusion

In this paper we present a database of length, width, and DNA content for mitotic chromosomes in vertebrate species, and a similar database of length and DNA content for flowering plants. We find simple power-law scaling relationships amongst these quantities. Notably, vertebrate and flowering plant chromosomes show nearly identical length scaling, despite their evolutionary divergence 1.6 billion years ago (Wang et al., 1999). This suggests that the scaling is rooted in a conserved feature of chromosome condensation and/or mitosis.

In this paper we also present one possible explanation for the observed scaling laws. Approximating the chromatid as a simple elastic rod, the cross sectional area will regulate its resistance to elongation. Since viscous drag forces are expected to scale with length, the cross section is expected to increase in parallel. Like all biological scaling arguments, the consistency between prediction and observation remains tentative without detailed measurements for each step in the argument. As of this writing, more information is needed on the relationship between chromatid width and stretching modulus, and also on the drag forces exerted on a chromatid by the adjacent spindle microtubules.

It should be noted that our hypothesized explanation for the scaling laws is not mutually exclusive with other approaches. In particular, we offer no speculations about the molecular mechanisms that match the cross section of mitotic chromosomes to their length. This may be compared to Brahmachari and Marko (2019), who use results from polymer physics to predict a length scaling exponent close to that found here. This provides an interesting and complementary perspective.

It is also interesting to consider how the results of this paper might differ for other eukaryotic clades. When looking beyond angiosperms and vertebrates, mitosis exhibits a wide degree of ultrastructural variability, including variation in the amount of chromatin condensation, the persistence or disintegration of the nuclear envelope, and variation in the geometry of the spindle (Heath, 1980). As discussed above, budding yeast shows no evidence of rodlike chromatin during mitosis, and the spindle is qualitatively smaller and simpler. Budding yeast also exhibits so-called “closed mitosis”, which means the nuclear envelope remains intact throughout cytokinesis.

Our scaling laws for mitotic chromosomes are not an isolated result. Besides prior scaling analyses for chromosomes, reviewed above, there is also a broader field of cell organelle scaling. It has been known for decades that nuclear size is positively correlated with C-value in eukaryotes (Baetcke et al., 1967; Maul and Deaven, 1977). More recently, attention has focused on the scaling properties of the nucleolus and the spindle (Hara and Kimura, 2013; Crowder et al., 2015; Uppaluri et al., 2016). While some progress has been made connecting the underlying molecular biology to various scaling results (Levy and Heald, 2012; Lacroix et al., 2018), much more remains to be done.

Lastly, we find that the range of chromosome lengths in a karyotype is typically larger in vertebrates than in flowering plants. We offer no conjectures here, but it is perhaps worth noting that flowering plants do not have centrioles, and that spindle MTs nucleate in a broad zone surrounding the condensing chromosomes (Yamada and Goshima, 2017). Whether this difference in spindle geometry has a relationship with karyotype, or whether one of the many other distinctions between plant and vertebrate cells is key, remains an open question.

## Data Availability Statement

The original contributions presented in the study are included in the article/Supplementary Material, further inquiries can be directed to the corresponding author.

## Author Contributions

EK planned the research, performed the statistical analysis, and wrote the manuscript. All authors contributed to the database.

## Conflict of Interest

The authors declare that the research was conducted in the absence of any commercial or financial relationships that could be construed as a potential conflict of interest.
