# Supplementary material for: Scaling Laws for Mitotic Chromosomes
Source: Front Cell Dev Biol. 2021 Jun 11;9:684278. doi: 10.3389/fcell.2021.684278 (PMC8262490; doi:10.3389/fcell.2021.684278)
Supplement: Supplementary file 3 [file Data_Sheet_1.PDF]

Supplemental Information for "Scaling Laws for Mitotic Chromosomes"

Authors: Eric M. Kramer, P. A. Tayjasanant, and Bethan Cordone

Contents:

Legend for Supplemental Table S1

Legend for Supplemental Table S2

Unified Reference List for the databases.

Legend for Supplemental Table S1. Database of information for mitotic chromosomes in vertebrate species. The table contains 19 columns. In order, these are a species index (assigned alphabetically), the class, order, family, genus, and species of the organism, a common name if available, the haploid genome size 1C, the average chromosome DNA content ($c_{avg} = 4C/2n$), the estimated DNA content of the longest chromosome in the karyotype [$c_{max} = (4C)(L_{max}/L_{tot})$], the source used for the 1C value (most values come from the animal genome size database of {Gregory, 2019 #1753}; in cases where multiple C-values are available, the median was used), the chromosome number 2n, length of the longest chromosome in the karyotype L_{max} (note the use of "um" for micron throughout), the average length of the chromosomes ($L_{avg} = L_{tot}/2n$), the diploid total chromosome length L_{tot}, source(s) used for the length and width of the chromosomes (in entries with two sources, one provided the distribution of relative chromosome lengths and the other provided one or more absolute lengths), the chromosome width w, the estimated volume of the longest chromosome [$V = 2\pi(L_{max})(w/4)^2$], and the estimated DNA density of the longest chromosome (c_{max}/V).

Legend for Supplemental Table S2. Database of information for mitotic chromosomes in angiosperm species. The table contains 14 columns. In order, these are a species index (assigned alphabetically), the clade, family, genus, and species of the organism, the haploid genome size 1C, the average chromosome DNA content ($c_{avg} = 4C/2n$), the estimated DNA content of the longest chromosome in the karyotype [$c_{max} = (4C)(L_{max}/L_{tot})$], the source used for the 1C value (most values come from the genome size database of {Leitch, 2019 #1754}; in cases where multiple C-values are available, the median was used), the chromosome number 2n, length of the longest chromosome in the karyotype L_{max} (note the use of "um" for micron throughout), the average

length of the chromosomes ($L_{avg} = L_{tot}/2n$), the diploid total chromosome length L_{tot}, and the source used for the length of the chromosomes.

Unified Reference List

(All citations that appear in Supplemental Tables S1 and S2.)

Aguiar-Jr, O., Lima, A. P., Giaretia, A. A. and Recco-Pimentel, S. M. (2002). Cytogenetic analysis of four poison frogs of the Epipedobates genus (Anura: Dendrobatidae). *Herpetologica* 58: 293-303.

Alvarez, M. C., Cano, J. and Thode, G. (1980). DNA Content and Chromosome Complement of Chromis Chromis (Pommacentridae, Perciformes). *Caryologia* 33: 267-274.

Ambastha, H. N. S. (1956). Cytological Investigations in Phalaris. *Genetica* 28: 64-98.

Aprea, G., Odierna, G., Andreone, F., Glaw, F. and Vences, M. (2007). Karyological evolution and systematics of Malagasy microhylid frogs. *Zoologischer Anzeiger* 246: 23-41.

Arnason, U. (1974b). Comparative chromosome studies in Pinnipedia. *Hereditas* 76: 179-226.

Arnason, U. (1981). Banding studies on the gray and sperm whale karyotypes. *Hereditas* 95(2): 277-281.

Asahida, T., Ida, H. and Hayashizaki, K.-i. (1995). Karyotypes and cellular DNA contents of some sharks in the order Carcharhiniformes. *Japan. J. Ichthyol.* 42: 21-26.

Asghari-Zakaria, R. (2007). Karyological Studies in Two Natural Populations of Boissiera squarossa. *International Journal of Agriculture & Biology* 9: 779-781.

Ayonoadu, U. W. U. (1974). Nuclear DNA Variation in Phaseolus. *Chromosoma* 48: 41-49.

Badr, F. M. and Badr, R. S. (1970). The somatic chromosomes of a wild population of rats: numerical polymorphism. *Chromosoma* 30(4): 465-475.

Bailey, P. C. (1949). Differential Chromosome Segments in Trillium erectum L. *Bulletin of the Torrey Botanical Club* 76: 319-336.

Bailey, P. C. (1951). A Study of the Chromosome Morphology of Some Species of Trillium. *Bulletin of the Torrey Botanical Club* 78: 324-330.

Bailey, P. C. (1954). A Further Study of the Chromosome Morphology of Some Species of Trillium. *Bulletin of the Torrey Botanical Club* 81: 68-75.

Baker, R. J. and Bleier, W. J. (1971). Karyotypes of bats of the subfamily Carolliinae (Mammalia; Phyllostomatidae) and their evolutionary implications. *Experientia* 27(2): 220-222.

Barat, A. and Khuda-Bukhsh, A. R. (1984). Karyomorphology of a sea-frog Tetraodon fluviatilis (Tetraodontidae,

Pisces). *Current Science* 53: 1108-1109.

Barsacchi-Pilone, G., Batistoni, R., Andronico, F., Vitelli, L. and Nardi, I. (1986). Heterochromatic DNA in Triturus (Amphibia, Urodela). I. A satellite DNA component of the pericentric C-bands. *Chromosoma* 93(5): 435-446.

Bhan, A., Langer, A. and Koul, A. K. (1989). Genetic Diversity among Plantagos IX. A nucleolar trisomic in Plantago Iagopus L. *Cytologia* 54: 729-736.

Blanco, A. and Perrino, P. (1974). Analisi citologica di alcune specie del genere Vicia. *Giornale botanico italiano* 108: 123-133.

Botschantzeva, Z. P. (1982). *Tulips: Taxonomy, Morphology, Cytology, Phytogeography and Physiology*, A. A. Balkema, Rotterdam.

Brown, N. P., Bromage, N. R., Penman, D. J. and Shields, R. I. (1997). The karyotype of the Atlantic halibut, Hippoglossus hippoglossus (Linnaeus). *Aquaculture Research* 28: 489-491.

Capanna, E., Conti, L. and De Renzis, G. (1968b). I Cromosomi di Barbastella Barbastellus (Mammalia — Chiroptera). *Caryologia* 21: 137-145.

Capanna, E. and Romanini, M. G. M. (1971). Nuclear DNA Content and Morphology of the Karyotype in Certain Palearctic Microchiroptera. *Caryologia* 24: 471-482.

Chen, C. (1969). The Somatic Chromosomes of Maize. *Canadian Journal of Genetics and Cytology* 11: 752-754.

Chiarelli, B. (1962a). Comparative Morphometric Analysis of Primate Chromosomes. I. The Chromosomes of Anthropoid Apes and of Man. *Caryologia* 15: 99-121.

Chiarelli, B. (1962b). Comparative Morphometric Analysis of Primate Chromosomes. II. The Chromosomes of the Genera Macaca, Papio, Theropithecus and Cercocebus. *Caryologia* 15: 401-420.

Chiarelli, B. (1963). Comparative Morphometric Analysis of Primate Chromosomes. III. The Chromosome of the Genera Hylobates, Colobus and Presbytis. *Caryologia* 16: 637-648.

Chiarelli, B., Ferrantelli, O. and Cucchi, C. (1969). The Caryotype of Some Teleostea Fish Obtained by Tissue Culture in vitro. *Experientia* 25.

Choudhury, R. C., Prasad, R. and Das, C. C. (1982). Karyological Studies in Five Tetraodontiform Fishes from the Indian Ocean. *Copeia* 1982: 728-732.

Chu, E. H. Y. and Giles, N. H. (1957). A Study of Primate Chromosome Complements. *The American Naturalist* 91: 273-282.

Comings, D. E. and Berger, R. O. (1969). Gene products of Amphiuma: an amphibian with an excessive amount of DNA. *Biochem Genet* 2(4): 319-333.

Constantinidis, T., Kamari, G. and Phitos, D. (1997). A cytological study of 28 phanerogams from the mountains of SE Sterea Ellas, Greece. *Willdenowia* 27: 121−142.

Contreras, L. C., Torres-Mura, J. C. and Spotorno, A. E. (1990). The largest known chromosome number for a mammal, in a South American desert rodent. *Experientia* 46(5): 506-508.

Coonen, L. P. (1939). The Chromosomes of Ranunculus. *American Journal of Botany* 26: 49-58.

Corcoran, M., F. and Travis, J. (1980). A Comparison of the Karyotypes of the Frogs Rana areolata, Rana sphenocephala, and Rana pipiens. *Herpetologica* 36: 296-300.

Crippa, M. (1964). The mouse karyotype in somatic cells cultured in vitro. *Chromosoma* 15: 301-311.

Dahlgren, G. (1991). Karyological investigations in Ranunculus subg. Batrachium (Ranunculaceae) on the Aegean islands. *Plant Systematics and Evolution* 177: 193-211.

Dallai, R. and Talluri, M. V. (1969). A Karyological Study of Three Species of Scincidae (Reptilia). *Chromosoma* 27: 86-94.

Das, A. B. and Mallick, R. (1989). Variation in Karyotype and Nuclear DNA Content in Different Varieties of Foeniculum vulgare Mill. *Cytologia* 54: 129−134.

De Boer, L. E. M. (1973). Cytotaxonomy of the Lorisoidea (Primates: Prosimii). I. Chromosome Studies and Karyological Relationships in the Galagidae. *Genetica* 44: 155-193.

De Boer, L. E. M. (1973b). Cytotaxonomy of the Lorisoidea (Primates: Prosimii). II. Chromosome studies in the Lorisidae and Karyological Relationships within the Superfamily. *Genetica* 44: 330-367.

De Boer, L. E. M. (1974). Cytotaxonomy of the Platyrrhini (Primates). *Genen Phaenen* 17: 1-115.

de Oliveira, E. H., Neusser, M., Figueiredo, W. B., Nagamachi, C., Pieczarka, J. C., Sbalqueiro, I. J., Wienberg, J. and Müller, S. (2002). The phylogeny of howler monkeys (Alouatta, Platyrrhini): reconstruction by multicolor cross-species chromosome painting. *Chromosome Res* 10(8): 669-683.

De Smet, W. H. O. (1978). The Chromosomes of 11 Species of Chelonia (Reptilia). *Acta zoologica et pathologica antverpiensia* 70: 15-34.

De Smet, W. H. O. (1978b). The Chomosomes of 23 Species of Snakes. *Acta zoologica et pathologica Antverpiensia* 70: 85-118.

De Smet, W. H. O. (1981). Description of the orcein stained karyotypes of 36 lizard species (Lacertilia, Reptilia) belonging to the families Teiidae, Scincidae, Lacertidae, Cordylidae and Varanidae (Autarchoglossa). *Acta zoologica et pathologica Antverpiensia* 76: 73-118.

Di Berardino, M. A. (1962). The Karyotype of Rana pipiens and Investigration of its Stability during Embryonic Differentiation. *Developmental biology* 5: 101-126.

Diosdado, J. C. and Pastor, J. (1993). Karyological studies of some orophyte taxa of the genus Ranunculus L. from the Iberian Peninsula. *Botanical Journal of the Linnean Society* 111: 23-35.

Donahue, W. H. (1974). A karyotypic study of three species of Rajiformes (Chondrichthyes, Pisces). *Can J Genet Cytol* 16(1): 203-211.

Donnelly, G. M. and Sparrow, A. H. (1965). Mitotic and meiotic chromosomes of Amphiuma. *Journal of Heredity* 56: 91-98.

Du, B. and Wang, D. (2006). C-values of seven marine mammal species determined by flow cytometry. *Zoolog Sci* 23(11): 1017-1020.

Dvorak, F. and Dadakova, B. (1984). Chromosome Counts and Chromosome Morphology of Some Selected Species. *Folia geobotanica et phytotaxonomica* 19: 41-74.

Dzhemilev, A., Bologovskaya, Z. M. and Machavariani, M. G. (1973). Comparative study of the chromosome sets of two species of macaque. *Tsitologiia* 15: 751-761.

Evans, L. E. (1962). Karyotype Analysis and Chromosome Designations for Diploid Agropyron Elongatum (Host) P.B. *Canadian Journal of Genetics and Cytology* 4: 267-271.

Falistocco, E. and Piccirilli, M. (1989). The Basic Karyotype of Lotus tenuis C-banding and Feulgen Studies. *Annals of Botany* 63: 401−404.

Fujiwara, I. (1956). Karyotype analysis in Plantago II. *Jap. Jour. Genet.* 31: 184-191.

Ghigliotti, L., Fevolden, S.-E., Cheng, C.-H. C., Babiak, I., Detta, A. and Pisano, E. (2012). Karyotyping and cytogenetic mapping of Atlantic cod (Gadus morhua Linnaeus, 1758). *Animal Genetics* 43: 746-752.

Ghimire, B. K., Yu, C. Y., Kim, H. J. and Chung, I. M. (2012). Karyotype and nucleic acid content in Zantedeschia aethiopica Spr. and Zantedeschia elliottiana Engl. *African Journal of Biotechnology* 11: 11604-11609.

Ghosh, A. and Datta, A. K. (2006). Karyotyping of Nigella sativa L. (Black Cumin) and Nigella damascena L. (Love-in-a- mist) by Image Analyzing System. *Cytologia* 71(1): 1-4.

Giorgi, B. and Bozzini, A. (1969). Karyotype Analysis in Triticum: III — Analysis of the Presumed Diploid Progenitors of Polyploid Wheats. *Caryologia* 22:3: 279-288.

Gitai, J., Paule, J., Zizka, G., Schulte, K. and Benko-Iseppon, A. M. (2014). Chromosome numbers and DNA content in Bromeliaceae: additional data and critical review. *Botanical Journal of Linnean Society* 176: 349-368.

Goepfert, D. (1974). Karyotypes and DNA Content in Species of Ranunculus L. and Related Genera. *Bot. Notiser* 127: 464-489.

Green, D. M., Wasserman, A. O. and Bogart, J. P. (1981). Karyotypes of the Frogs, Rana septentrionalis and R. virgatipes. *Copeia* 1981: 879-882.

Gregory, T. R. (2019). "Animal Genome Size Database." from www.genomesize.com.

Greilhuber, J. and Speta, F. (1978). Quantitative Analyses of C-Banded Karyotypes, and Systematics in the Cultivated Species of the Scilla siberica Group (Liliaceae). *Plant Systematics and Evolution* 129: 63-109.

Grozeva, N. (2007). Mediterranean chromosome number reports (1623-1630). *Flora Mediterranea* 17: 299-307.

Grutzner, F., Lutjens, G., Rovira, C., Barnes, D. W., Ropers, H.-H. and Haaf, T. (1999). Classical and molecular cytogenetics of the pufferfish Tetraodon nigroviridis. *Chromosome Research* 7: 655-662.

Guerra, M., Dos Santos, K. G., Barros, E. S. A. E. and Ehrendorfer, F. (2000). Heterochromatin banding patterns in Rutaceae-Aurantioideae--a case of parallel chromosomal evolution. *Am J Bot* 87(5): 735-747.

Gupta, P. P. (1977). Cytogenetics of Aquatic Ornamentals: III. Karyology of Victoria Amazonica Sowerby. *Current Science* 46: 574-575.

Haertel, J. D., Owczarzak, A. and Storm, R. M. (1974). A Comparative Study of the Chromosomes from Five Species of the Genus Rana (Amphibia: Salientia). *Copeia* 1974: 109-114.

Haga, T. (1934). The Comparative Morphology of the Chromosome Complement in the Tribe Parideae. *Botany* 3: 1-32.

Hamerton, J. L., Fraccaro, M., De Carli, L., Nuzzo, F., Klinger, H. P., Hulliger, L., Taylor, A. and Lang, E. M. (1961). Somatic chromosomes of the gorilla. *Nature* 192: 225-228.

Hamilton, A. E. and Buettner-Janusch, J. (1977). Chromosomes of Lemuriformes

III. The Genus Lemur: Karyotypes of Species, Subspecies, and Hybrids. *Ann N Y Acad Sci* 293: 125-159.

Hammar, B. (1966). The Karyotypes of Nine Birds. *Hereditas* 55: 367-385.

Hammar, B. (1970). The karyotypes of thirty-one birds. *Hereditas* 65: 29-58.

Hara, Y., Adachi, K., Kagohashi, S., Yamagata, K., Tanabe, H., Kikuchi, S., Okumura, S.-I. and Kimura, A. (2016). Scaling relationship between intra-nuclear DNA density and chromosomal condensation in metazoan and plant. *Chromosome Science* 19: 43-49.

Hartley, S. E. and Horne, M. T. (1984). Chromosome relationships in the genus Salmo. *Chromosoma* 90(3): 229-237.

Hennen, S. (1964). The Karyotype of Rana Sylvatica and its Comparison with the Karyotype of Rana Pipiens. *J Hered* 55: 124-128.

Hirahara, S. and Tatuno, S. (1967). Cytologial Studies on Narcissus I. Karyotype and nucleolinus of Narcissus jonquilla. *Cytologia* 32: 553-559.

Hohmann, N., Wolf, E. M., Lysak, M. A. and Koch, M. A. (2015). A Time-Calibrated Road Map of Brassicaceae Species Radiation and Evolutionary History. *Plant Cell* 27(10): 2770-2784.

Hsu, T. C. and Arrighi, F. E. (1971). Distribution of Constitutive Heterochromatin in Mammalian Chromosomes. *Chromosoma* 34: 243-253.

Hu, F. R., Liu, H. H., Wang, F., Bao, R. L. and Liu, G. X. (2015). Root tip chromosome karyotype analysis of hyacinth cultivars. *Genet Mol Res* 14(3): 10863-10876.

Hungerford, D. A., Chandra, H. S. and Snyder, R. L. (1967). Somatic Chromosomes of a Black Rhinoceros (Diceros bicornis Gray 1821). *American Naturalist* 101: 357-358.

Hynniewta, M., Malik, S. K. and Rao, S. R. (2011). Karyological studies in ten species of Citrus(Linnaeus, 1753) (Rutaceae) of North-East India. *Comp Cytogenet* 5(4): 277-287.

Jarrell, G. H. and Arnason, U. (1981). Banded karyotypes of a belukha whale, Delphinapterus leucas. *Hereditas* 95(1): 37-41.

Jaylet, A. (1966). Le Caryotype De L'amphibien Urodele Euproctus Asper (Duges). *Chromosoma* 18: 79-87.

Jha, T. B. (2019). Karyotype Analysis from Aerial Roots of Piper nigrum Based on Giemsa and Fluorochrome Banding. *Cytologia* 84(4): 313-317.

Jha, T. B., Mahanti, A. and Ghorai, A. (2015). Karyotype analysis of Indian lentils through EMA based Giemsa staining. *Caryologia* 68: 280-288.

Jones, R. N. and Brown, L. M. (1976). Chromosome Evolution and DNA Variation in Crepis. *Heredity* 36(1): 91-104.

Kamel, E. A. (1999). Karyological Studies on Some Taxa of the Genus Vicia L. (Fabaceae). *Cytologia* 64: 441-448.

Kaneko, K. (1968). Cytological Studies on Some Species of Hosta II. Karyotypes of H. rectifolia, H. opipara, H. venusta, H. amanuma and H. capitata. *The Botanical Magazine, Tokyo* 81: 267-277.

Kessous, A., Caussinus, H., Jaylet, A. and Beetschen, J. C. (1968). Essai d'analyse biometrique du caryotype de l'Amphibien Urodele Salamandra salamandra L. *Chromosoma* 23: 324-332.

Khuda-Bukhsh, A. R. and Barat, A. (1987). Chromosomes in Fifteen Species of Indian Teleosts (Pisces). *Caryologia* 40: 131-144.

Kochjarova, J., Valachovic, M., Bures, P. and Mraz, P. (2006). The genus Cochlearia L. (Brassicaceae) in the Eastern Carpathians and adjacent area. *Botanical Journal of the Linnean Society* 151: 355–364.

Kong, H.-Z. (2000). Karyotypes of Sarcandra Gardn. and Chloranthus Swartz (Chloranthaceae) from China. *Botanical Journal of the Linnean Society* 133: 327-342.

Konishi, T., Funamoto, T., Smirnov, S. V. and Damdinsuren (2011). Chromosome studies in seven species of Allium (Alliaceae sensu stricto) in Mongolian and Russian Altai. *Chromosome Botany* 6: 53-60.

Korth, J. W. and Fitzsimons, J. M. (1987). Karyology of Three Species of Eastern North American Atherinid Fishes. *Copeia* 1987: 505-509.

Koul, A. K. and Wafai, B. A. (1980). Chromosome Polymorphism and Nucleolar Organization in Some Species of Fritillaries Linn. *Cytologia* 45: 675-682.

Kulshreshtha, V. B. and Gupta, P. K. (1981). Cytogenetic Studies in the Genus Helianthus L. II. Karyological studies in twelve species. *Cytologia* 46: 279-289.

Lacerda, M. M., Silva, J. C., Vieira, A. T. and Clarindo, W. R. (2019). Cytogenetic Characterization of Passiflora megacoriacea K.Port.-Utl. Employing Image Cytometry. *Cytologia* 84: 353-357.

Lakshmanan, P. S., Van Laere, K., Eeckhaut, T., Van Huylenbroeck, J., Van Bockstaele, E. and Khrustaleva, L. (2015). Karyotype analysis and visualization of 45S rRNA genes using fluorescence in situ hybridization in aroids (Araceae). *Comparative Cytogenetics* 9: 145-160.

Las Penas, M. L., Urdampilleta, J. D., Lopez-Carro, B., Santinaque, F., Kiesling, R. and Bernardello, G. (2014). Classical and molecular cytogenetics and DNA content in Maihuenia and Pereskia (Cactaceae). *Plant Systematics and Evolution* 300: 549-558.

LeGrande, W. H. (1975). Karyology of Six Species of Louisiana Flatfishes (Pleuronectiformes: Osteichthyes). *Copeia* 1975: 516-522.

Leitch, I. J., Johnston, E., Pellicer, J., Hidalgo, O. and Bennett, M. D. (2019). "Angiosperm DNA C-values database (release 9.0)." from https://cvalues.science.kew.org/.

Lim, K.-B., Wennekes, J., de Jong, J. H., Jacobsen, E. and van Tuyl, J. M. (2001). Karyotype analysis of Lilium longiflorum and Lilium rubellum by chromosome banding and fluorescence in situ hybridisation. *Genome* 44: 911-918.

Liming, S., Yingying, Y. and Xingsheng, D. (1980). Comparative cytogenetic studies on the red muntjac, Chinese muntjac, and their F1 hybrids. *Cytogenetics and Cell Genetics* 26: 22-27.

Macgregor, H. C. and Jones, C. (1977). Chromosomes, DNA Sequences, and Evolution in Salamanders of the Genus Aneides. *Chromosoma* 63: 1-9.

Marasek, A. and Orlikowska, T. (2003). KARYOLOGY OF NINE LILY GENOTYPES. *ACTA BIOLOGICA CRACOVIENSIA Series Botanica* 45: 159-168.

Marks, G. E. (1957). The cytology of Oxalis dispar (Brown). *Chromosoma* 8(6): 650-670.

Martin, E., Unal, M., Dogan, B., Altinordu, F., Sefali, A. and Kaya, A. (2016). Karyotype Analyses of the Genus Matthiola (Brassicaceae) in Turkey. *Cytologia* 81: 53-60.

Martin, P. G. and Hayman, D. L. (1967). Quantitative comparisons between the karyotypes of Australian marsupials from three different superfamilies. *Chromosoma* 20(3): 290-310.

Martonfiova, L. (2013). A method of standardization of chromosome length measurement. *Caryologia* 66:4: 304-312.

Marutani, M., Sheffer, R. D. and Kamemoto, H. (1993). Cytological Analysis of Anthurium andraeanum (Araceae), Its Related Taxa and Their Hybrids. *American Journal of Botany* 80: 93-103.

Masoud, S., Shirin, Z.-F., Shadi, K. and Bahram, Z. (2002). Karyotypic study in some Iranian species and populations of Tulipa L. (Liliaceae). *Caryologia* 55:1: 81-89.

Matoba, H., Mizutani, T., Nagano, K., Hoshi, Y. and Uchiyama, H. (2007). Chromosomal study of lettuce and its allied species (Lactuca spp., Asteraceae) by means of karyotype analysis and fluorescence in situ hybridization. *Hereditas* 144: 235-243.

Mehra, P. N. and Pandita, T. K. (1984). Cytological studies of some Helobiales of Kasmir Himalayas I. Family Alismataceae. *Cytologia* 49: 295-304.

Mercado-Ruaro, P. and Delgado-Salinas, A. (1998). Karyotypic studies on species of Phaseolus (Fabaceae: Phaseolinae). *Am J Bot* 85(1): 1.

Mercado-Ruaro, P. and Delgado-Salinas, A. (2009). Karyotypic analysis in six species of Phaseolus L. (Fabaceae). *Caryologia* 62: 167-170.

Merritt, J. F. and Lacks, G. D. (1991). Karyology Of The Black Sea Bass, Centropristis striata. *Journal of the Elisha Mitchell Scientific Society* 107: 75-78.

Michaelis, A. and Rieger, R. (1959). Strukturheterozygotie bei Vicia faba. *Dur Zuchter* 29: 354-361.

Micheli, G., Luzzatto, A. R., Carrì, M. T., de Capoa, A. and Pelliccia, F. (1993). Chromosome length and DNA loop size during early embryonic development of Xenopus laevis. *Chromosoma* 102(7): 478-483.

Miyaki, K., Tabeta, O. and Kayano, H. (1995). Karyotypes in Six Species of Pufferfishes Genus Takifugu (Tetraodontidae, Tetraodontiformes). *Fisheries Science* 61: 594-598.

Moler, P. E. and Kezer, J. (1993). Karyology and Systematics of the Salamander Genus Pseudobranchus (Sirenidae). *Copeia* 1993: 39-47.

Monkheang, P., Chaveerach, A., Sudmoon, R. and Tanee, T. (2016). Karyotypic features including organizations of the 5S, 45S rDNA loci and telomeres of Scadoxus multiflorus (Amaryllidaceae). *Comp Cytogenet* 10: 637-646.

Moore, R. (1965). A Biometric Analysis of the Chromosomes of the Marsupials - Macropus major, Macropus rufus and Potorous tridactylis. *Cytogenetics* 4: 145-156.

Morescalchi, A., Odierna, G. and Olmo, E. (1977). Karyological relationships between the Cryptobranchid salamanders. *Specialia* 15: 1579-1581.

Morescalchi, A., Odierna, G. and Olmo, E. (1979). Karyology of the primitive salamanders, family Hynobiidae. *Experientia* 35(11): 1434-1436.

Mudry, M. D., Nieves, M. and Steinberg, E. R. (2015). Cytogenetics of Howler Monkeys. *Howler Monkeys: Adaptive Radiation, Systematics, and Morphology*. M. K. Martin, A. G. Paul, C.-O. Liliana, U. Bernardo and Y. Dionisios, Springer, New York**:** 85-105.

Nardi, I., Ragghianti, M. and Mancino, G. (1973). Banding patterns in newt chromosomes by the Giemsa stain. *Chromosoma* 40(4): 321-331.

Nayyar, R. P. (1964). Karyotype Studies in Seven Species of Cyprinidae. *Genetica* 35: 95-104.

Nieves, M., De Oliveira, E. H., Amaral, P. J., Nagamachi, C. Y., Pieczarka, J. C., Mühlmann, M. C. and Mudry, M. D. (2011). Analysis of the heterochromatin of Cebus (Primates, Platyrrhini) by micro-FISH and banding pattern comparisons. *J Genet* 90(1): 111-117.

Noleto, R. B., Vicari, M. R., Cestari. Marta, M. and Artoni, R. F. (2012). Variable B chromosomes frequencies between males and females of two species of pufferfishes (Tetraodontiformes). *Reviews in Fish Biology and Fisheries* 22: 343-349.

Ohno, S. and Atkin, N. B. (1966). Comparative DNA values and chromosome complements of eight species of fishes. *Chromosoma* 18: 455-466.

Ohno, S., Muramoto, J., Stenius, C., Christian, L., Kittrell, W. A. and Atkin, N. B. (1969). Microchromosomes in holocephalian, chondrostean and holostean fishes. *Chromosoma* 26(1): 35-40.

Oud, J. L. and Schuring, F. (1987). Computer assisted karyotype analysis of Pyrrhopappus carolinianus. *Genetica* 74: 211-217.

Ozturk, M., Martin, E., Dinc, M., Duran, A., Ozdemir, A. and Cetin, O. (2009). A Cytogenetical Study on Some Plants Taxa in Nizip Region (Aksaray, Turkey). *Turkish Journal of Biology* 33: 35-44.

Pal, T., Ghosh, S., Mondal, A. and De, K. K. (2016). Evaluation of genetic diversity in some promising varieties of lentil using karyological characters and protein profiling. *J Genet Eng Biotechnol* 14(1): 39-48.

Palma-Rojas, C., Jara-Seguel, P. and Brand, E. V. (2007). Karyological studies in Chilean species of Bomarea and Leontochir (Alstroemeriaceae). *New Zealand Journal of Botany* 45: 299-303.

Palomino, G., Martinez, J., Romero, P., Barba-Gonzalez, R. and Rodriguez-Garay, B. (2017). Nuclear genome size and karyotype analysis of Agave angustifolia Haw. “Cimarron” and “Lineño” (Asparagales, Asparagaceae). *Caryologia* 70.

Park, J. Y., Kim, K., Sohn, H., Kim, H. W., An, Y. R., Kang, J. H., Kim, E. M., Kwak, W., Lee, C., Yoo, D., Jung, J., Sung, S., Yoon, J. and Kim, H. (2018). Deciphering the evolutionary signatures of pinnipeds using novel genome sequences: The first genomes of Phoca largha, Callorhinus ursinus, and Eumetopias jubatus. *Sci Rep* 8(1): 16877.

Paszko, B. (2006). A critical review and a new proposal of karyotype asymmetry indices. *Plant Systematics and Evolution* 258: 39-48.

Pegington, C. and Rees, H. (1970). Chromosome weights and measures in the Triticinae. *Heredity* 25: 195-205.

Pellicer, J., Fay, M. F. and Leitch, I. J. (2010). The largest eukaryotic genome of them all? *Botanical Journal of Linnean Society* 164: 10-15.

Pellicer, J., Kelly, L. J., Leitch, I. J., Zomlefer, W. B. and Fay, M. F. (2014). A universe of dwarfs and giants: genome size and chromosome evolution in the monocot family Melanthiaceae. *New Phytol* 201: 1484-1497.

Peruzzi, L., Carta, A. and Altinordu, F. (2017). Chromosome diversity and evolution in Allium (Allioideae, Amaryllidaceae). *Plant Biosystems* 151:2: 212-220.

Pierozzi, N. I. (2011). Karyotype and Nor-Banding of Mitotic Chromosomes of Some Vitis L. Species. *Jaboticabal SP* E: 564-570.

Pierre, P. M., Sousa, S. M., Davide, L. C., Machado, M. A., Viccini, L. F., Hai-xia, Y., Ding, C.-B., Yang, R.-W., Zhang, L., Zhou, Y.-H. and Li, Y. (2011). Karyotype analysis, DNA content and molecular screening in Lippia alba (Verbenaceae)

Karyomorphology of some taxa of Paris (Melanthiaceae) from Sichuan province, China. *An Acad Bras Cienc* 83(3): 993-1006.

Piovesan, A., Pelleri, M. C., Antonaros, F., Strippoli, P., Caracausi, M. and Vitale, L. (2019). On the length, weight and GC content of the human genome. *BMC Res Notes* 12(1): 106.

Poggio, L., Wulff, A. F. and Hunziker, J. H. (1986). CHROMOSOME SIZE, NUCLEAR VOLUME and DNA CONTENT IN BULNESIA (ZYGOPHYLLACEAE). *Darwiniana* 27 (1-4): 25-38.

Popov, P. and Dimitrov, B. (1996). Comparative karyotype analysis of 24-chromosome Rana species (R. cbensinensis and R. arvalis, Amphibia, Anura). *Caryologia* 49: 163-173.

Potter, I. C., Robinson, E. S. and Walton, S. M. (1968). The mitotic chromosomes of the lamprey Mordacia mordax (Agnatha: Petromyzonidae). *Experientia* 24(9): 966-967.

Potter, I. C. and Rothwell, B. (1970). The mitotic chromosomes of the lamprey, Petromyzon marinus L. *Experientia* 26(4): 429-430.

Praca-Fontes, M. M., Carvalho, C. R. and Ronildo, W. R. (2014). Karyotype revised of Pisum sativum using chromosomal DNA amount. *Plant Syst Evol* 300: 1621−1626.

Qu, L., Xue, L., Xing, G., Zhang, Y., Chen, J., Zhang, W. and Lei, J. (2018). Karyotype analysis of eight wild Tulipa species native to China and the interspecific hybridization with tulip cultivars. *Euphytica* 214:65.

Ragghianti, M., Bucci-Innocenti, S. and Mancino, G. (1978). Karyology of the Carpathian Newt Triturus Montandoni and Cytotaxonomic Considerations on the Species Group T. Vulgaris (Urodela: Salamandridae). *Caryologia* 31:2: 243-256.

Raicu, P., Vranceanu, V., Mihailescu, A., Popescu, C. and Motz, M. K. (1976). Research of the Chromosome Complement in Helianthus L. Genus. *Caryologia* 29: 307-316.

Ranney, T. G., Ryan, C. F., Deans, L. E. and Lynch, N. P. (2018). Cytogenetics and Genome Size Evolution in Illicium L. *Hortscience* 53(5): 620-623.

Ray-Chaudhuri, S. P., Sharma, T., Pathak, S. and Singh, L. (1969). Chromosomes and the Karyotype of the Pangolin, Manis pentadactyla L. (Pholidota-Mammalia). *Specialia* 25: 1167-1168.

Reig, O. A. and Bianchi, N. O. (1969). The occurrence of an intermediate didelphid karyotype in the short-tailed opossum (genus Monodelphis). *Experientia* 25(11): 1210-1211.

Reig, O. A., Gardner, A. L., Bianchi, N. O. and Patton, J. L. (1977). The chromosomes of the Didelphidae (Marsupialia) and their evolutionary significance. *Biological Journal of the Linnean Society* 9: 191-216.

Rejon, M. R. and Sanudo, A. (1976). Estudios Cariologicos en Especies EspañOlas del Orden Liliales.

I. Allium, Lapiedra, Narcissus. *Lagascalia* 6: 225-238.

Renzoni, A. and Vegni-Talluri, M. (1966). The Karyograms of Some Falconiformes and Strigiformes. *Chromosoma* 20: 133-150.

Rivlin, K. A., Dale, G. and Rachlin, J. W. (1986). Karyotypic Analysis of Three Species of Cardinal Fish (Apogonidae) and Its Implications for the Taxonomic Status of the Genera Apogon and Phaeoptyx. *Annals new york academy of sciences*: 211-213.

Rothfels, K. and Heimburger, M. (1968). Chromosome Size and DNA Values in Sundews (Droseraceae). *Chromosoma* 25: 96-103.

Rothfels, K., Sexsmith, E., Heimburger, M. and Krause, M. O. (1966). Chromosome size and DNA content of species of Anemone l. and related genera (Ranunculaceae). *Chromosoma* 20: 54-74.

Rudak, E. and Callan, H. G. (1976). Differential Staining and Chromatin Packing of the Mitotic Chromosomes of the Newt Triturus cristatus. *Chromosoma* 56: 349-362.

Sanso, M. A. and Hunziker, J. H. (1998). Karyological studies in Alstroemeria and Bomarea (Alstroemeriaceae). *Hereditas* 129: 67-74.

Sarbhoy, R. K. (1980). Karyological Studies in the Genus Phaseolus, Linn. *Cytologia* 45: 363-373.

Saroniya, R. K., Nagpure, N. S., Saksena, D. N., Kushwaha, B. and Kumar, R. (2013). Cytotaxonomic Studies in Four Species of Genus Puntius (Hamilton, 1822) from Central India. *National Academy of Science Letters* 36(4): 411-418.

Schmid, M. (1978). Chromosome Banding in Amphibia: I. Constitutive Heterochromatin and Nucleolus Organizer Regions in Bufo and Hyla. *Chromosoma* 66: 361-388.

Schmid, M. (1980). Chromosome Banding in Amphibia: V. Highly Differentiated ZW/ZZ Sex Chromosomes and Exceptional Genome Size in Pyxicephalus adspersus (Anura, Ranidae). *Chromosoma* 80: 69-96.

Schmid, M., Vitelli, L. and Batistoni, R. (1987). Chromosome banding in amphibia. XI. Constitutive heterochromatin, nucleolus organizers, 18S + 28S and 5S ribosomal RNA genes in Ascaphidae, Pipidae, Discoglossidae and Pelobatidae. *Chromosoma* 95(4): 271-284.

Schreeb, K. H., Groth, G., Sachsse, W. and Freundt, K. J. (1993). The karyotype of the zebrafish (Brachydanio rerio). *J Exp Anim Sci* 36(1): 27-31.

Schwarzacher-Robinson, T. (1986). SC-Formation, and Karyotype Structure in Diploid Paeonia tenuifolia and Tetraploid P. officinalis. *Plant Systematics and Evolution* 154: 259-274.

Schweizer, D., Ambros, P., Grundler, P. and Varga, F. (1987). Attempts to Relate Cytological and Molecular Chromosome Data of Arabidopsis Thaliana to its Genetic Linkage Map. *Arabidopsis Information Service* 25: 27-34.

Sessions, S. K. (1980). Evidence for a Highly Differentiated Sex Chromosome Heteromorphism in the Salamander Necturus maculosus (Rafinesque). *Chromosoma* 77: 157-168.

Sessions, S. K. (1982). Cytogenetics of Diploid and Triploid Salamanders of the Ambystoma jeffersonianum Complex. *Chromosoma* 84: 599-621.

Sessions, S. K. and Kezer, J. (1987). Cytogenetic evolution in the plethodontid salamander genus Aneides. *Chromosoma* 95: 17-30.

Sessions, S. K., Leon, P. E. and Kezer, J. (1982b). Cytogenetics of the Chinese Giant Salamander, Andrias davidianus (Blanchard): The Evolutionary Significance of Cryptobranchoid Karyotypes. *Chromosoma* 86: 341-357.

Seto, T., Matsui, M. and Kawakami, E. (1986). The Giemsa stained and C-banded Karyotypes of Hynobius tsuensis and H. leechii (Amphibia, Urodela)*. *Japanese Journal of Herpetology* 11(3): 137-144.

Seto, T., Utsunomiya, Y. and Utsunomiya, T. (1983). Karyotypes of Two Representative Species of Hynobiid Salamanders, Hynobius nebulosus (Schiegel) and Hynobius naevius (Schiegel). *Proc. of the Japan Acad.* 59: 231-235.

Sharma, A. and Datta, K. B. (1961). A Cytological Study to work out the Trend of Evolution in Aglaonema and Richardia. *Caryologia* 14: 439-454.

Sharma, A. and Toor, I. S. (1980). Cytogenetical Investigations into Some Garden Ornamentals II. The genus Aloe L. *Cytologia* 45: 515-532.

Sharma, P. K., Koul, A. K. and Langer, A. (1984). Genetic Diversity among Plantagos II. Karyotype of Plantago Ianceolata L. with special emphasis on nucleolar chromosomes. *Cytologia* 49: 351-357.

Shaver, E. L. (1962). The chromosomes of the opossum, Didelphis virginiana. *Can J Genet Cytol* 4: 62-68.

Shen, B. (1990). Karyotype analysis of Illicium verum Hook. *Journal of Southwest Forestry College* 10: 56-59.

Shindo, K. and Kamemoto, H. (1963). Karyotype Analysis of Some Species of Phalaenopsis. *Cytologia* 28: 390-398.

Sikka, K. and Sharma, A. K. (1979). Chromosome Evolution in Certain Genera of Brassiceae. *Cytologia* 44: 467-477.

Siljak-Yakovlev, S., Peccenini, S., Muratovic, E., Zoldos, V., Robin, O. and Valles, J. (2003). Chromosomal differentiation and genome size in three European mountain Lilium species. *Plant Systematics and Evolution* 236: 165-173.

Silva, J. C., Carvalho, C. R. and Clarindo, W. R. (2018). Updating the maize karyotype by chromosome DNA sizing. *PLoS One* 13(1): e0190428.

Sinclair, E. A., Murch, A. R., Di Renzo, M. and Palermo, M. (2000). Chromosome morphology in Gilbert's potoroo, Potorous gilbertii (Marsupialia : Potoroidae). *Australian Journal of Zoology* 48: 281-287.

Smyth, D. R., Kongsuwan, K. and Wisudharomn, S. (1989). A survey of C-band patterns in chromosomes of Lilium (Liliaceae). *Plant Systematics and Evolution* 163: 53-69.

Southern, D. I. (1967). Species Relationships in the Genus Tulipa. *Chromosoma* 23: 80-94.

Srivastava, S. and Srivastava, H. M. (2000). Cytological and Karyotypic Studies in Four Beta Species. *Journal of Sugar Beet Research* 37: 135-142.

Stanyon, R., Consigliere, S., Bigoni, F., Ferguson-Smith, M., O'Brien, P. C. and Wienberg, J. (2001). Reciprocal chromosome painting between a New World primate, the woolly monkey, and humans. *Chromosome Res* 9(2): 97-106.

Stingo, V. (1979). New Developments in Vertebrate Cytotaxonomy: Ii. The Chromosomes of the Cartilaginous Fishes. *Genetica* 50,3: 227-239.

Stingo, V. and Rocco, L. (2001). Selachian cytogenetics: a review. *Genetica* 111(1-3): 329-347.

Stone, D. E. and Freeman, J. L. (1968). Cytotaxonomy of Illicium Floridanum and I. Parviflorum (Illiciaceae). *Journal of the Arnold Arboretum* 49: 41-51.

Subramanian, D. and Munian, M. (1988). Cytotaxonomical Studies in South Indian Araceae. *Cytologia* 53: 59-66.

Sumner, A. T. (1991). Scanning electron microscopy of mammalian chromosomes from prophase to telophase. *Chromosoma* 100: 410-418.

Sun, B., Tian, Y., Zhang, F. and Tang, H. (2018). Chromosomal Karyotype Analysis of Four Varieties of Green Petioles Leaf Beet. *IOP Conf. Series: Materials Science and Engineering* 452: 022073.

Sybenga, J. (1959). SOME SOURCES OF ERROR IN THE DETERMINATION OF CHROMOSOME LENGTH. *Chromosoma* 10: 355-364.

Tanaka, N. (1981). Studies on Chromosome Arrangement in Some Higher Plants III. Haplopappus gracilis (2n=4) and Crepis capillaris (2n=6). *Cytologia* 46: 545-559.

Tanaka, R. (1967). A Comparative Karyotype Analysis in Haplopappus gracilis (2n=4) and H. ravenii (2n=8). *Cytologia* 32: 542-552.

Taper, L. J. and Grant, W. F. (1973). The Relationship Between Chromosome Size and DNA Content in Birch (Betula) Species. *Caryologia* 26: 263-273.

Terziiski, D. and Dimitrov, B. (1983). Karyotype Analysis in Vicia Hirsuta (L.) S. F. Gray and Vicia Meyeri Boiss. *Caryologia* 36: 345-354.

Thode, G., Cano, J. and Alvarez, M. C. (1983). A Karyological Study on Four Species of Mediterranean Gobiid Fishes. *Cytologia* 48: 131-138.

Trivedi, R. N. and Roy, R. P. (1970). Cytological Studies in Cucumis and Citrullus. *Cytologia* 35: 561-569.

Tymowska, J. (1973). Karyotype analysis of Xenopus tropicalis Gray, Pipidae. *Cytogenet Cell Genet* 12(5): 297-304.

Tymowska, J. and Fischberg, M. (1973b). Chromosome complements of the genus Xenopus. *Chromosoma* 44(3): 335-342.

Tzanoudakis, D. (1983). Karyotypes of four wild Paeonia species from Greece. *Nordic Journal of Botany* 3: 307-318.

Ullerich, F. H. (1970). DNA content and chromosome structure in Amphibia. *Chromosoma* 30(1): 1-37.

Utech, F. H. and Kawano, S. (1976). Biosystematic Studies in Erythronium (Liliaceae-Tulipeae) III. Somatic karyotype analysis of E. japonicum Decne. *Cytologia* 41: 749-755.

Vaio, M., Nascimento, J., Mendes, S., Ibiapino, A., Felix, L. P., Gardner, A., Emshwiller, E., Fiaschi, P. and Guerra, M. (2018). Multiple karyotype changes distinguish two closely related species of Oxalis (O. psoraleoides and O. rhombeo-ovata) and suggest an artificial grouping of section Polymorphae (Oxalidaceae). *Botanical Journal of Linnean Society* 188: 269-280.

Veleba, A., Smarda, P., Zedek, F., Horova, L., Smerda, J. and Bures, P. (2017). Evolution of genome size and genomic GC content in carnivorous holokinetics (Droseraceae). *Ann. Bot.* 119: 409-416.

Vitturi, R., Carbone, P., Catalano, E. and Macaluso, M. (1984). Chromosome Polymorphism in Gobius Paganellus, Linneo 1758 (Pisces, Gobiidae). *Biol Bull* 167(3): 658-668.

Warren, W. C., Kuderna, L., Alexander, A., Catchen, J., Pérez-Silva, J. G., López-Otín, C., Quesada, V., Minx, P., Tomlinson, C., Montague, M. J., Farias, F. H. G., Walter, R. B., Marques-Bonet, T., Glenn, T., Kieran, T. J., Wise, S. S., Wise, J. P., Jr., Waterhouse, R. M. and Wise, J. P., Sr. (2017). The Novel Evolution of the Sperm Whale Genome. *Genome Biol Evol* 9(12): 3260-3264.

Winterfeld, G., Becher, H., Voshell, S., Hilu, K. and Roser, M. (2018). Karyotype evolution in Phalaris (Poaceae): The role of reductional dysploidy, polyploidy and chromosome alteration in a wide-spread and diverse genus. *PLoS ONE* 13: e0192869.

Wurster, D. H. and Benirschke, K. (1967). Chromosome Studies in Some Deer, the Springbok, and the Pronghorn, with Notes on Placentation in Deer. *Cytologia* 32: 273-285.

Wurster, D. H. and Benirschke, K. (1970). Indian muntjac, Muntiacus muntjak: a deer with a low diploid chromosome number. *Science* 168: 1364-1366.

Yamamoto, K. (1973). Karyotaxonomical Studies on Vicia I. On the Karyotype and Character of Some Annual Species of *Vicia*. *Japanese Journal of Genetics* 48: 315-327.

Yao, J.-L., Rowland, R. E. and Cohen, D. (1994). Karyotype studies in the genus Zantedeschia (Araceae). *South African Journal of Botany* 60: 4-7.

Yesil, Y. and Ozhatay, F. N. (2014). Morphological, anatomical and karyological investigations on the genus Paris in Turkey. *Biological Diversity and Conservation* 7: 57-69.

Zanandrea, S. J. G. and Capanna, E. (1964). Contributo alla cariologia del genere Lampetra. *Bolletino di zoologia* 31: 669-677.

Zheng, J., Nakata, M., Uchiyama, H., Morikawa, H. and Tanaka, R. (1991). Giemsa C-banding Patterns in Several Species of Phaseolus L. and Vigna Savi, Fabaceae. *Cytologia* 56: 459-466.

Zhihai, H., Jiang, X., Shuiming, X., Baosheng, L., Yuan, G., Chaochao, Z., Xiaohui, Q., Wen, X. and Shilin, C. (2016). Comparative optical genome analysis of two pangolin species: Manis pentadactyla and Manis javanica. *Gigascience* 5(1): 1-5.

Zonneveld, B. J. M. (2009). The systematic value of nuclear genome size for ''all'' species of Tulipa L. (Liliaceae). *Plant Syst Evol* 281: 217-245.
